# A Study on the Attitude and Perception of Bachelor of Medicine and Bachelor of Surgery (MBBS) Students Regarding Post-MBBS Compulsory Service Bond in Uttar Pradesh, India

**DOI:** 10.7759/cureus.44682

**Published:** 2023-09-04

**Authors:** Harish Chandra Tiwari, Richa Mishra, Yogendra Singh, Imran Ahmed Khan, Dhirendra K Srivastava

**Affiliations:** 1 Community Medicine, Baba Raghav Das Medical College, Gorakhpur, IND; 2 Community Medicine, Mahamaya Rajkiya Allopathic Medical College, Ambedkar Nagar, IND; 3 Anatomy, Baba Raghav Das Medical College, Gorakhpur, IND

**Keywords:** medical education, service bond, rural health services, perception, medical students, attitude

## Abstract

Background

To increase the availability of doctors in the public healthcare delivery system, the state government of Uttar Pradesh, India, has implemented a two-year compulsory service bond since 2018. Students of the 2018 batch are going to complete their Bachelor of Medicine, Bachelor of Surgery (MBBS) in 2023 and are supposed to serve through this bond. There are many dilemmas in the minds of medical students regarding their compulsory service bond. Hence, there is a need to know their attitude and perceptions regarding the compulsory service bond. This study was conducted to assess the attitude and perception of undergraduate medical students toward compulsory service bonds.

Methods

This was a mixed-method study conducted in July-September 2022 among undergraduate medical students at Baba Raghav Das Medical College, Gorakhpur, Uttar Pradesh, India. For quantitative data, a structured questionnaire was developed using Google Forms (Google LLC, Mountain View, California, United States) and circulated via WhatsApp (Meta Platforms, Inc., Menlo Park, California, United States) through the random sampling method. Focused group discussions were carried out to collect the qualitative data.

Result

Regarding the compulsory service bond after MBBS, 100 (31.8%) medical students were found to be interested and 56 (17.8%) were disinterested. The majority (n=158; 50.4%) of participants were neutral, while 278 (88.6%) medical students perceived it as an opportunity to help poor people. Higher possibilities of social recognition and respect were some noticeable perceptions of 243 (77.4%) MBBS students. Lack of confidence to tackle serious cases without a senior doctor’s supervision was perceived as an important hurdle by 286 (91%) participants. Non-availability of advanced medical facilities, issues like the safety of doctors, and the lack of availability of electricity, roads, and infrastructure were also perceived as hurdles.

Conclusions and recommendations

Students perceived the compulsory service bond as an opportunity if met with certain conditions like a transparent method of posting and basic facilities or an incentive for accommodation and transportation. The compulsory service bond for addressing the shortfall of doctors in the public healthcare delivery system may be more effective if these hurdles are corrected and certain opportunities are met, as mentioned in the present study. This will help the government move smoothly towards achieving Universal Health Coverage (UHC).

## Introduction

World Health Organization (WHO) has advocated a desirable doctor-population ratio of 1:1,000 to provide adequate health services to a defined population [1]. Several low- and middle-income countries including India are facing a scarcity of trained health workforce in their healthcare delivery system [2]. A major part of healthcare services is being provided by private practitioners. Since the majority of trained doctors prefer to work in urban areas, healthcare services in rural areas are mainly provided by government doctors [3].

There is a huge shortage of qualified doctors for the rural healthcare delivery system in Uttar Pradesh, India. To increase the availability of doctors in the public healthcare delivery system, the state government of Uttar Pradesh has implemented a two-year compulsory service bond. This was implemented in 2018 for all MBBS students admitted to government medical colleges in Uttar Pradesh [4]. The first batch of these students is going to complete their MBBS in 2023. There are many queries and confusion in the minds of medical students regarding their compulsory service bond. Strong opposition has also been faced against compulsory service bond implementation in certain states [5].

This compulsory service bond will be fruitful only if the doctors going to serve under this bond have a positive attitude and perception regarding it. So, there is a considerable need to outline the attitude and perception of medical students regarding compulsory service bonds. The present survey was conducted to assess their attitude and perception towards rural medical service bonds and identify the associated factors responsible for preference or non-preference and the reasons associated with their choices.

## Materials and methods

Study design

This was a mixed-method study carried out from July 1, 2022, to September 30, 2022. For quantitative data collection, a web-based online survey using Google Forms (Google LLC, Mountain View, California, United States) was carried out among MBBS students of Baba Raghav Das Medical College, Gorakhpur, Uttar Pradesh, India. For qualitative data, focused group discussions (FDGs) were carried out.

Study tools

For quantitative data collection, a structured questionnaire was used. It was pilot-tested on a sample of 25 medical students who were not included in the final study. Necessary amendments were made as per the findings of the pilot study. The final questionnaire was reviewed by two independent experts not involved in the study and Cronbach’s alpha was 0.79 showing good internal consistency. The content validity index for individual items of the questionnaire was calculated to check the validity of the questionnaire. For qualitative data collection, FGDs were done and for it, pen and paper were used for recording of responses.

Methodology and data collection

For quantitative data collection, the study questionnaires were sent to eligible participants through WhatsApp (Meta Platforms, Inc., Menlo Park, California, United States) with a brief description of the study, indicating the voluntary nature of the survey, and a consent declaration, if they intended to participate. Follow-up messages were sent on the day after the first message, after one week, and before survey closure.

For qualitative data collection, FGDs involving four batches of MBBS students were done. Five FGDs per batch, each involving 15-20 students were carried out. So, a total of 20 FGDs were done to cover all four batches. FGDs were carried out in the community hall of Baba Raghav Das Medical College in the evening. In each FGD, one FGD leader and one observer participated. The FGD leader introduced the topic and facilitated the discussion among the participants. They also kept a check on the participant's deviation from the topic. The observer recorded the important findings of the discussion. In the end, the FGD leader closed the discussion and the content of the recorded discussion was rechecked by both the FGD leader and the observer that all the important findings had been recorded.

Statistical analysis

The data obtained from the Google Form responses were downloaded as an Excel sheet and imported to and analyzed using IBM SPSS Statistics for Windows, Version 23.0 (Released 2015; IBM Corp., Armonk, New York, United States). Descriptive statistics were performed. Only salient findings from the analysis have been described in this article. A content and thematic analysis was done by integrating all the recorded responses of the 20 FGDs. An inductive approach was used to develop themes. Codes used in the thematic analysis were: interest in working in rural areas, helping poor people, getting real learning experience, safety issues, problems regarding conveyance and accommodation, lack of senior guidance, and weightage/incentives in the service.

## Results

A total of 337 responses were obtained from participants. Twenty-three responses were incomplete and they were removed from the final analysis. Thus, 314 responses were included in the final analysis. Sociodemographic characteristics are compiled in Table 1.

**Table 1:** Sociodemographic profile of study participants (N=314)

Sociodemographic profile		Frequency	Percentage
Gender	Male	205	65.3
	Female	109	34.7
Residential background	Rural	137	43.6
	Urban	177	56.4
Medium of schooling	Hindi	81	25.8
	English	233	74.2
Type of schooling	Government	87	27.7
	Private	227	72.3
Education of father	Illiterate	4	1.3
	Primary (Up to Class-5)	15	4.8
	High school (Up to Class 10)	34	10.8
	Intermediate (Class 10+2)	50	15.9
	Graduate and above	211	67.2
Education of mother	Illiterate	48	15.3
	Primary (Up to Class-5)	32	10.2
	High school (Up to Class 10)	43	13.7
	Intermediate (Class 10+2)	57	18.2
	Graduate and above	134	42.7
Occupation of father	None	6	1.9
	Unskilled worker (e.g. Laborer)	5	1.6
	Skilled worker (it requires some skills e.g. Electrician, Mechanics, Carpenter etc.)	7	2.2
	Agriculture	68	21.7
	Business	58	18.5
	Government Job	117	37.3
	Private Job	53	16.9
Occupation of mother	Housewife	266	84.7
	Skilled worker (it requires some skill e.g. mechanics, electrician, carpenter etc.)	2	0.6
	Business	1	0.3
	Government Job	29	9.2

**Figure 1:**
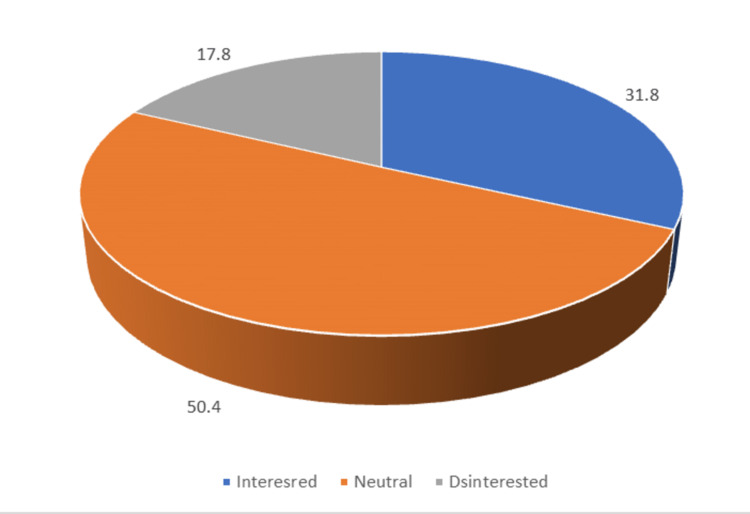
Attitude of participants about compulsory service bond after graduation

MBBS students from batch 2018 to 2021 participated in the study. About two-thirds (n=205; 65.3%) were male and the rest 109 (34.7%) were female. One hundred and seventy-seven (56.4%) of the participants were from urban backgrounds and 137 (43.6%) were from rural backgrounds. Students who have done their schooling from government schools were 87 (27.7%) and 227 ( 72.3%) were from private schools. The majority of the participants' fathers (n=211; 67.2%) studied up to graduation and above and 134 (42.7%) mothers were graduates. Regarding occupation, 117 (37.3%) fathers were in government jobs, 68 (21.7%) were in agriculture, 58 (18.5%) were in business, and 53 (16.9%) were in private jobs. Two hundred and sixty-six (84.7%) of their mothers were housewives. When asked about the compulsory rural service after graduation, 100 (31.8%) were found interested, 56 (17.8%) were found disinterested, and 158 (50.4%) were neutral (Figure 1).

MBBS students had some positive perceptions regarding the compulsory service bond, as summarized in Table 2. This was looked on as an opportunity to help poor patients by the majority of students (n= 278; 88.6%). Similarly, this was also looked upon as an opportunity to know more about rural health issues (n=275; 87.6%). At the same time, 276 (87.9%) participants expected weightage in postgraduate entrance examinations because of this, and 243 (77.4%) expected higher social recognition and respect.

**Table 2:** Perceived benefits of compulsory service bond

Sr. No.	Perceived benefits of compulsory service bond.	Number	Percentage
1	Opportunity to help poor people/patients	278	88.6
2	Weightage may be given in PG entrance examinations	276	87.9
3	Opportunity to know more about rural health issues	275	87.6
4	Higher possibility of social recognition & respect	243	77.4
5	Less workload so more free time for studies	169	53.8
6	Greater possibility of private practice	162	51.6
7	Less interference of administration	157	50

This survey also draws attention to some possible hurdles (Table 3) during compulsory service bonds. The unavailability of advanced medical facilities (n=300; 95.6%), lack of senior support and supervision to tackle serious cases (n=286; 91%), and lack of confidence/experience to work in rural areas (n=283; 90.7%) were among the top drawbacks perceived by MBBS students.

**Table 3:** Perceived hurdles during compulsory service bond

Sr. No.	Perceived hurdles in compulsory service bond	Agree	
		Number	Percentage
1	Non-availability of advanced medical facilities in rural areas	300	95.6
2	Non-availability of senior support & supervision	286	91
3	Lack of confidence/experience to work in rural areas	283	90.7
4	Lack of good schools for children (in case they have children)	282	89.8
5	Lack of access to amenities	280	89.2
6	Safety of doctors	278	88.6
7	Limited opportunity for professional development	264	84.1
8	Poor living conditions	264	84.1
9	Difficulty if spouse is working in an urban area	257	81.8
10	Less income/earning	241	76.8
11	Interference of local people	225	71.7
12	Lack of social prestige/ recognition	195	62.1

After completion of the survey and data analysis, MBBS students were invited for FGD on a predefined day. FGD revealed three important themes regarding the compulsory service bond, summarized in Table 4.

**Table 4:** Thematic analysis of focused group discussion outlined following themes NEET (PG): National Eligibility Entrance Test (Postgraduate)

Theme 1	Perceived opportunities regarding compulsory service bond.
	It must be appreciated, as health facilities in rural and remote areas greatly affect people’s lives. Interested in working in rural areas. I want to help poor people and those who can’t afford high-cost treatment in private hospitals. Working in both urban and rural areas will be a real learning experience.
Theme 2	Perceived hurdles regarding compulsory service bond.
	The safety of doctors by rule of law and political interference during duty will be problem. There may be problem regarding electricity, road and infrastructure. Availability of drugs and consumables as per the expectation of people would be difficult.
Theme 3	Perceived as opportunities if met with certain conditions.
	Availability to take guidance or advice from a senior/expert doctor by any method, as I don’t feel confident enough to treat every disease. A transparent method of posting for compulsory service bond must be outlined and communicated to us. The weightage of this service in the NEET (PG) entrance examination should be considered. Basic facilities or incentive for accommodation and transportation should be given.

## Discussion

In the present study, we found 100 (31.8%) participants were willing to do a compulsory service bond after completing MBBS. They were interested in working in rural areas and wanted to help poor people and perceived it as an opportunity. This finding is similar to a study done by Nallala et al., who found nearly 30% of participants were willing to work in rural areas [6]. Lal et al reported that medical students were found to prefer hospital-based clinical specialties and wanted to practice in urban locations [7]. A study by Gupta et al. reported that the majority of doctors were not interested in working in the government health system, much less in rural and remote locations [8]. 

Some medical educationists have proposed rural medical schools as a strategy to address the growing unwillingness of students for rural medical careers [9]. Mandatory rural service has its problems that must be addressed. Just making it mandatory is not enough; students’ apprehension must be addressed and their willingness to work should be encouraged [10]. Otherwise, the reluctance of the existing workforce itself will be a problem in providing services to the rural population. Kumar and Pal in their editorial also proposed a change in public health discourse in India in augmenting resources/strategy for recruitment and retaining medical doctors in rural areas [11]. 

In our study, certain hurdles in the compulsory service bond were pointed out by participants. Important hurdles included a lack of confidence or experience and the non-availability of senior/experienced doctors' support in the management of patients. Similar findings were also enumerated in a study conducted by Choudhary et al. [12]. A study by Singh conducted in India revealed that undergraduate medical students preferred to do postgraduate courses because they felt that they could not manage patients with the inadequate knowledge they acquired as undergraduates [13]. 

FGD outlined some conditions which when met could attract more doctors to serve in rural areas. Participants opined that availability to take guidance/advice from senior/expert doctors by any method, transparent method of posting for compulsory service bond, improving basic facilities or incentive for accommodation and transportation, and weightage of this service in National Eligibility Entrance Test (Postgraduate) (NEET (PG)) entrance may have a favorable impact in serving in rural areas. These findings are in line with some other studies in the literature [14,15]. WHO recommendations to improve the retention of healthcare workers in rural areas may contribute substantially to the goal of UHC to include rural people [16]. 

In the study by Goel, it has been observed that the doctors serve in rural areas for the completion of their rural bond and then shift back to urban areas [17].

Strengths and limitations

This pioneering study provides information regarding the possible challenges of doctors going to serve under a service bond. This might be helpful to decision-makers in implementing the compulsory service bond in a self-reliant environment. One of the limitations of our study is that it is based on students’ perceptions and opinions from a single institute before their real experience of the service bond; thus, further studies will be required involving doctors from different institutions after the completion of their service bond. Another limitation might be the online nature of data collection.

Recommendations

The compulsory service bond as a system for addressing the shortfall of doctors in the public healthcare delivery system may be more effective if the perceived hurdles are corrected and met with certain opportunities as mentioned in the present study. This will help the government to move smoothly towards achieving UHC.

## Conclusions

Regarding the compulsory service bond after MBBS, about one-third of MBBS students were interested and less than one-fifth were disinterested. Half of the participants were neutral. Some students want to work in rural areas and perceive it as an opportunity to help poor people. Lack of confidence to work in rural areas to tackle serious cases without senior support and supervision was perceived as among the top hurdles. The non-availability of advanced medical facilities, issues like the safety of doctors, and the lack of availability of electricity, roads, and infrastructure were perceived as hurdles. Students perceived the bond as an opportunity if met with certain conditions, like a transparent method of posting for compulsory service bonds and basic facilities or incentives for accommodation and transportation.
